# Detecting gene–gene interactions from GWAS using diffusion kernel principal components

**DOI:** 10.1186/s12859-022-04580-7

**Published:** 2022-02-01

**Authors:** Andrew Walakira, Junior Ocira, Diane Duroux, Ramouna Fouladi, Miha Moškon, Damjana Rozman, Kristel Van Steen

**Affiliations:** 1 BIO3 - Laboratory for Systems Genetics, GIGA-R Medical Genomics, University of Liège, Liège, Belgium; 2 Centre for Functional Genomics and Bio-Chips, Institute for Biochemistry and Molecular Genetics, Faculty of Medicine, University of Ljubljana, Ljubljana, Slovenia; 3 Faculty of Computer and Information Science, University of Ljubljana, Ljubljana, Slovenia; 4 BIO3 - Laboratory for Systems Medicine, Department of Human Genetics, KU Leuven, Leuven, Belgium

**Keywords:** Inflammatory bowel disease, Diffusion kernel principal components, Bivariate synergy, Spike and slab priors, Gene epistasis network

## Abstract

Genes and gene products do not function in isolation but as components of complex networks of macromolecules through physical or biochemical interactions. Dependencies of gene mutations on genetic background (i.e., epistasis) are believed to play a role in understanding molecular underpinnings of complex diseases such as inflammatory bowel disease (IBD). However, the process of identifying such interactions is complex due to for instance the curse of high dimensionality, dependencies in the data and non-linearity. Here, we propose a novel approach for robust and computationally efficient epistasis detection. We do so by first reducing dimensionality, per gene via diffusion kernel principal components (kpc). Subsequently, kpc gene summaries are used for downstream analysis including the construction of a gene-based epistasis network. We show that our approach is not only able to recover known IBD associated genes but also additional genes of interest linked to this difficult gastrointestinal disease.

## Introduction

It was Bateson [1] who first described epistasis as a biological process in which gene expression at one locus is suppressed by a gene at another locus. Several years later, Fisher came up with a non-equivalent definition of epistasis expressed in terms of deviations from a model of additive multiple effects regardless the scale (linear or logarithmic) [2]. In this work, we adopt the most commonly used reference to “epistasis”, as referring to any interaction between genes in which the contribution of one gene to a phenotype depends on genetic background. For more details about epistasis, what it means and does not mean, its analytic challenges and reproducibility concerns, we refer to [3] and more references in [4, 5]. Notably, epistasis research has evolved into a more general theory and application framework for the analysis of interactions across and between omics strata.

Focusing on epistasis detection with data collected in genome-wide association studies (GWAS) [6–8], there are still numerous hurdles that when not taken care of properly may decrease our belief in the results and may complicate their interpretation [4]. Examples include computational and statistical issues related to the high-dimensionality of GWAS data and the corresponding number of mutli-locus genotype combinations. Indeed, genome-wide Association Interaction Studies (GWAIS) involve hundreds of thousands of genetic markers (usually single nucleotide polymorphisms or SNPs) that need to be interrogated in pairs (or k-tuples). This makes correcting for multiple testing a daunting task. It is therefore not a surprise that the minority of epistasis detection methods aim for higher-order (more than k = 2) interactions. One example is BHIT [9], a Bayesian High-order Interaction Toolkit for detecting epistatic interactions among SNPs.

In order to deal with problems associated with high dimensional modeling and testing, some researchers have applied filtering approaches to identify and only include in the final analysis, SNPs that are most probable to be involved in interactions. For example, Hemani and colleagues [10] applied a two stage analysis process. In the first stage, an experimental threshold was determined and used to remove SNPs with significant additive or dominant effects leaving a smaller set of unique SNP pairs for the second stage of the analysis. This is different from early days GWAIS practices where only GWAS hits were considered for subsequent epistasis checks. These different practices can be explained by considering the statistical hypotheses underlying the epistasis study: detecting interactions above and beyond main effects or detecting multi-locus joint effects (for more details see; Van Steen and Moore [4]). Also Pecanka et al. [11] applied a two stage strategy to be able to maximize the chances for epistasis signal detection with a reduced set. The reduction was achieved by applying a two-locus independence test in cases only prior to epistasis screening in cases and controls jointly. Several years before, two-locus information had been used by Calle et al. [12], who identified potentially interacting genes using a synergy measure in stage one and applied a prototype Model-Based Multifactor Dimensionality Reduction technique (MB-MDR) on the reduced set in stage 2.

Generally, these and similar methods have been successful in some cases but may also suffer from epistasis detection power loss. Furthermore, they embed a degree of subjectivity due to the choice of filtering or dimensionality reduction technique; different choices often leading to quite different results [13]. Thresholds need to be selected that may not be driven by biology but may need to be informed by sample size. For instance, GenEpi employs a feature extraction process to extract promising SNPs within each gene using randomized machine learning techniques. The randomization, even though increasing the computational burden, was introduced to reduce the number of false positives. However, small sample sizes may lead to over-fitting in GenEpi and require stringent feature selection thresholds at the expense of false negatives and unstable sets of positive signals in replication data [14].

In this work, we propose a novel epistasis detection analysis workflow that (1) takes GWAS SNP data as input, (2) develops gene-level summaries via diffusion kernels on graphs, and (3) uses these summaries as new units in epistasis (gene-gene) interaction modelling. We illustrate the workflow using a Bayesian modelling framework and inflammatory bowel disease as case study.

## Materials and methods

### Data and data pre-processing

We used GWAS data on Inflammatory Bowel Disease (IBD) as part of the International IBD Consortium (www.ibdgenetics.org) and carried out quality control (QC) procedures as described in Ellinghaus et al. [15]. SNPs that were in Linkage Disequilibrium ($r^2 > 0.75$) were pruned out. Only common variants (minor allele frequency $> 5\%$) and those in Hardy-Weinberg equilibrium (*p* value $> 0.001$) were considered. Then, we focused on SNPs potentially relevant for IBD. Specifically, FUMA software [16] was used to create eQTL SNP to gene mapping that mapped a SNP to its target gene when the association *p* value was significant in the colon. In addition, specific for the purposes of GWAIS, extra QC implementations were made as described in [17], building on recommendations from [18]. After these QC steps, the data comprised 66,280 individuals (32,622 cases and 33,658 controls) and 4398 SNPs.

Furthermore, the dichotomous phenotype (IBD or not) was corrected for population structure using the top 7 principal component analysis [19]. As in [15], the top 7 principal components were used to capture population structure. Trait correction for confounders is often done in epistasis research, especially when the targeted modeling framework does not accommodate the inclusion of fixed explanatory variables.

**Fig. 1:**
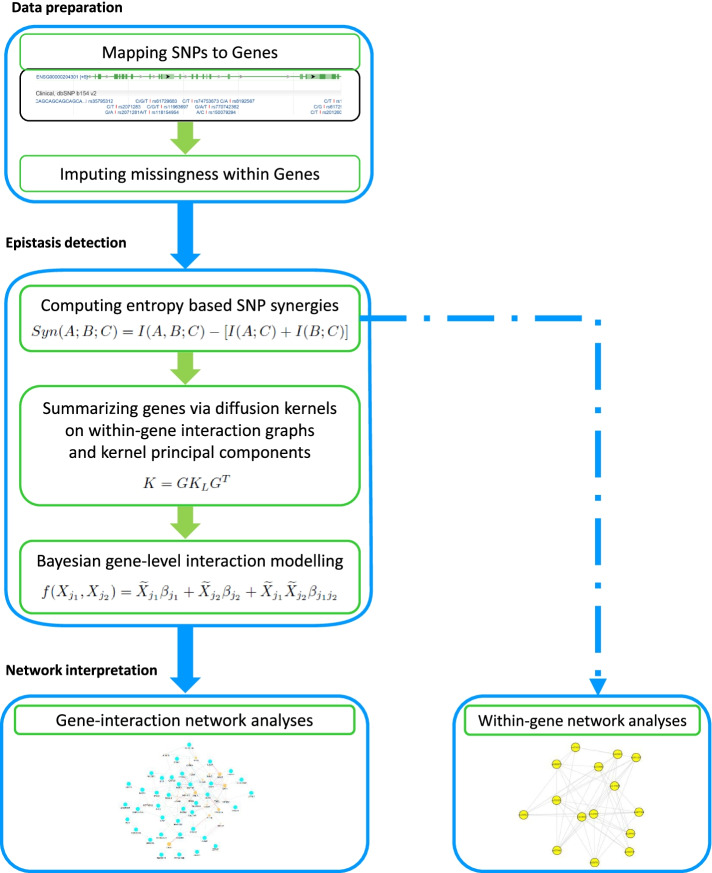
Novel epistasis detection workflow: SNPs are mapped to respective genes followed by imputation for missing genotypes. Interaction information between SNPs allocated to the same gene is used to compute diffusion kernels and graphical within-gene network structures. Data reduction via kernel principal component analysis gives gene summaries (representations) that are submitted to an epistasis detection model of choice (here, for illustration, a Bayesian model). Gene-gene interactions are graphically represented via a gene-level statistical epistasis network and interpreted

### Gene–gene interaction analysis workflow with diffusion kernel principal components

Our proposed epistasis detection strategy starts with SNP-to-gene annotation, which was carried out using the FUMA software [16] via eQTL mapping. This led to “gene files” with sets of SNPs. For those SNPs, missing genotypes were handled via *k*-nearest neighbour (*k*NN) imputation with $k = 10$ using the *knn*.*impute* function in the bnstruct package [20]. The average missingness rate per SNP was 0.062%. Subsequent steps are explained in more detail in the following subsections. The entire analysis workflow is depicted in Fig. 1. Unless mentioned otherwise, all following data analyses are performed in the statistical software R, version 3.5.1 [21].

#### Within-gene synergy

Bivariate synergy was used to construct SNP-based graphs within a gene and the resulting within-gene edge weights were used to obtain informed summaries of genes. We first discretized (binned) the population-structure corrected phenotype using k-means clustering based on the equal-width method implemeted in the *discretize* function in the Infotheo package [22, 23]. Such binning strategies overcome difficulties in information gain computations for continuous phenotypes and have been shown to be useful for interaction detection between pairs of genetic markers [24]. Second, we calculated bivariate synergy *Syn* between a pair of SNPs as described by [25, 26]. In particular, given two SNPs *A* and *B*, and the phenotype *C*, *Syn* was calculated as follows:

1 $$\begin{aligned} Syn(A;B;C) = I(A, B;C) - [I(A;C) + I(B;C)] \end{aligned}$$

where *Syn*(*A*; *B*; *C*) compares the joint contribution of SNPs *A* and *B* to the phenotype *C* with the additive contributions of the individual SNPs. The information gain *I*(*A*; *C*) about the phenotype *C* due to knowledge about SNP *A* and is defined as:

2 $$\begin{aligned} I(A;C)= & {} H(C) - H(C|A) \end{aligned}$$

3 $$\begin{aligned} I(A,B;C)= & {} H(C) - H(C|A,B) \end{aligned}$$

Here, *H*(*C*) denotes the entropy of *C* and *H*(*C*|*A*) (respectively *H*(*C*|*A*, *B*)) refers to the conditional entropy of *C* given knowledge of SNP *A* (and *B*). The entropy and conditional entropy of *C* are defined as:

4 $$\begin{aligned} H(C)= & {} \sum _{c} p(c)log\frac{1}{p(c)} \end{aligned}$$

5 $$\begin{aligned} H(C|A)= & {} \sum _{a,c} p(a,c)log\frac{1}{p(c|a)} \end{aligned}$$

with *p*(*c*) the probability that an individual has phenotype $C=c$. Likewise, *p*(*c*|*a*) is the probability of having phenotype $C=c$ given genotype *a* for SNP *A*.

#### Within-gene network analysis

For the construction of within-gene networks, SNPs were used as nodes and bivariate synergy between two SNPs provided edge weights. The “Maximum Relevance Minimum Redundancy” algorithm in the minet package in R [27] was used for meaningful node selection. In particular, the bivariate synergy matrices computed before were subjected to the *mrnet* function. The *mrnet* algorithm then computes a score that is used to rank the set of SNPs (vertices). For a particular target *Y* (each SNP is used as a target in turn), the algorithm starts by selecting the SNP $S={X_i}$ with the highest synergy with *Y*. Then SNP $X_j$ with high synergy with *Y* and low synergy with $X_i$ is selected. The algorithm updates *S*, the set of selected variables, by choosing the SNP:

6 $$\begin{aligned} X_j^{MRMR} = \underset{X_j \in V\backslash S}{{\text {argmax}}} (u_j - r_j) \end{aligned}$$

that maximises the score $s_j = u_j - r_j$ where $u_j$ is the relevance term $u_j = I(X_j;Y)$ and $r_j$ is the redundancy term:

7 $$\begin{aligned} r_j = \frac{1}{|S|}\sum _{X_{k}\in S} I(X_{j};X_{k}) \end{aligned}$$

The SNP network is then inferred by removing edges using an incremental search algorithm [27, 28]. This resulted in a reduced gene SNP set that was analysed with the igraph R package [29]. Several network properties were recorded for each within-gene network [30] including density and mean distance. A network’s density is defined as the ratio of actual versus potential connections. Mean distance is defined as the average of shortest paths between nodes. As a network connectivity measure, we chose transitivity i.e. capturing the tendency of network edges to form triangles via the ratio between the observed number of closed triplets and the maximum possible number of closed triplets in the network.

#### Diffusion kernel principal components

Diffusion kernels were constructed over genotypes based on matrix exponentiation as described by [31, 32]. Using the adjacency matrix with bivariate synergies (*Syn*) computed in “Within-gene synergy” section, as off-diagonal weights and zeroes on the diagonal the Laplacian for each gene’s graph *G* was defined as:

$$\begin{aligned} L_{ij}= {\left\{ \begin{array}{ll} W_{ij},&{} \text {for } i\ne j\\ - \sum _{l=1}^{n} W_{il}, &{} \text {for } i = j. \end{array}\right. } \end{aligned}$$

Here, *i* refers to $SNP_{i}$ and *j* to $SNP_{j}$, and $W_{ij} = Syn(SNP_i; SNP_j; C)$. With this Laplacian, the diffusion kernel is defined as

8 $$\begin{aligned} K_{L} = e^{\beta L} \end{aligned}$$

with $\beta$ is a parameter that regulates the degree of “diffusion”. Note that $K_{L}$ is a matrix exponential. A $\beta$ value of zero gives an identity diffusion kernel matrix. We generated 101 diffusion kernels $K_{L}$ for $\beta$ ranging from 0 and 10 (increments of 0.1) and took the average of the 101 $K_{L}$ to derive the final kernel matrix $K_{L}$.

Assuming *G* to be a $n \times p$ genotype matrix, with *n* individuals and *p* SNPs allocated to the same gene, and $G^T$ its transpose, the final gene-specific kernel of interest was defined as

9 $$\begin{aligned} K = G K_L G^T \end{aligned}$$

Notably, *K* is a $n \times n$ kernel matrix that has information about gene-level similarity between individuals, as well as joint effects of SNPs on the trait within each gene. We then centered each kernel matrix, performed an eigen decomposition using the RSpectra package [33], and extracted the first principal component as a gene summary. The obtained gene constructs were considered as new units of gene-level epistasis analyses.

#### Detecting gene–gene interactions

An abundance of epistasis detection approaches with GWAS data exist. The vast majority of these approaches model or test for interactions at the SNP-level, assuming discrete data as input for their algorithms. In contrast, our proposed workflow assumes non-discrete input data i.e., one continuous variable per gene. The epistasis detection problem with GWAS data is consequently turned into a statistical problem that aims to at least capture, but preferentially detect, interactions between pairs of variables. Here, as an illustration, we used the Bayesian semi-parametric regression approach of [34] to infer non-linear gene-gene interactions, implemented in the NLinteraction R package. As gene explanatory variables for an individual *i*, we used the first kernel principal component $X_{ij_1}$ for each gene $j_1$ (see previous section). Genes and gene-gene interactions were selected on the basis of a so-called Posterior Inclusion Probability (PIP). This is a value for each effect of interest that indicates how likely it is to be included in the true model. Lesaffre and Lawson reported that PIP can “replace classical *p* values” [35]. See [36–38] for more information and application of PIP.

In particular, main effects and 2-way interaction effects were modelled as follows, exemplified for two genes $j_1$ and $j_2$: The main effects of gene 1 ($j_1$) were modelled as in Eq. (11).

10 $$\begin{aligned} f(X_{j_1}) = {\widetilde{X}}_{j_1}\beta _{j_1} \end{aligned}$$

and the interaction effect between gene 1 and gene 2 were modelled as in Eq. (12).

11 $$\begin{aligned} f(X_{j_1}, X_{j_2})= & {} {\widetilde{X}}_{j_1}\beta _{j_1} + {\widetilde{X}}_{j_2}\beta _{j_2} \nonumber \\&+\, {\widetilde{X}}_{j_{1}} {\widetilde{X}}_{j_{2}}\beta _{j_{1}j_{2}} \end{aligned}$$

with ${\widetilde{X}}_{j_1} = g_{1}(X_{j_1})$ and ${\widetilde{X}}_{j_2} = q_{1}(X_{j_2})$, where *g*(.) and *q*(.) are natural basis functions of $X_{j_1}$ and $X_{j_2}$ respectively and ${\widetilde{X}}_{j_{1}j_{2}} = g_{1}(X_{j_1})q_{1}(X_{j_2})$ the basis expansion of the interaction between $X_{j_1}$ and $X_{j_2}$, as explained in [34].

The complete model formulation makes the following assumptions for the response $Y_i$ for individual *i*, $i:1 \ldots n$, *n* the number of individuals:

12 $$\begin{aligned}&Y_{i} \sim Normal(f(X_{i}, \sigma ^2))\\&f(X_{i}) = \sum _{h = 1}^k f^{(h)}(X_{i}) \\&f^{(h)}(X_{i}) = \sum _{j_{1} = 1}^p {\widetilde{X}}_{ij1}\beta _{j_{1}}^{(h)} + \sum _{j_{1}=2}^p \sum _{j_{2} \le j_{1}} {\widetilde{X}}_{ij_{1}j_{2}}\beta _{j_{1}j_{2}}^{(h)} \end{aligned}$$

Here, *p* refers to the number of genes, and *k* is a number sufficiently large such that all exposure effects can be captured by the model.

At the core of Bayesian modelling lies choosing a prior distribution, here for $\beta _{j}$ and $\beta _{j_1j_2}$ in order to enforce sparsity. For this analysis, we kept the default settings of the authors: spike and slab priors were used to shrink parameters to zero hereby reducing the dimensionality of the data. In particular:

13 $$\begin{aligned} P(\beta _{S}^{(h)}|\zeta ) = \left( 1-\prod _{j \in s} \zeta _{jh} \right) \delta _{0} + \left( \prod _{j \in s} \zeta _{jh} \right) \psi (\beta _{S}^{(h)}) \end{aligned}$$

where *S* is a subset of 1,2, ..., *p* and $P(\zeta _{jh}) = \tau _{h}^{\zeta _{jh}}(1 - \tau _{h})^{1 - \zeta _{jh}}1(A_{h} \not \subset A_{m} \forall m$ or $A_{h} = \{\})$ where $A_{h} = \{j:\zeta _{jh} = 1\}$ and $\zeta = \{\zeta _{jh}\}$, a matrix of binary indicators of which genes and interactions are included in the *h*th function in the model. Spike was considered to be a point mass at zero i.e. $P(\beta = 0) > 0$, and slab, $\psi ()$, a multivariate distribution centered at **0** with covariance $\Sigma _{\beta }$ (estimated by empirical Bayes) as a diagonal matrix with $\sigma ^2\sigma ^2$ on the diagonals. Both $\tau _{h}$ and $\Sigma _{\beta }$ control the amount of shrinkage. In order to assess convergence, we kept track of $\sigma ^2$ as shown in the trace plot (see Additional file 3: Figure S3a).

#### Gene-level epistasis visualisation and gene annotation

Genes with a marginal PIP greater than zero and unique genes involved in interactions with a PIP greater than zero were retained for network analyses. In particular, the unique sets of genes thus obtained were submitted to GeneMANIA to visualize and interpret inter-connectivity between identified genes [39]. Furthermore, unique genes involved in gene-gene interactions ($PIP>0$) were propagated over a inBio network [40]. The inBio network is a protein–protein interaction network that integrates different studies and interaction types into a single evidence based integrated score for each gene/protein pair. To decipher the roles of highlighted genes as well as their involvement in IBD pathology, the gene annotation data base DAVID [41, 42] and DisGeNET [43, 44] were interrogated.

## Results

SNPs were physically mapped to respective genes yielding a total of 878 genes (“gene files”). These genes were spread across 22 chromosomes (no sex chromosomes). We successfully calculated kernels for 496 genes. The computation of kernels was restricted to genes with three or more SNPs mapped to the respective gene.

**Fig. 2:**
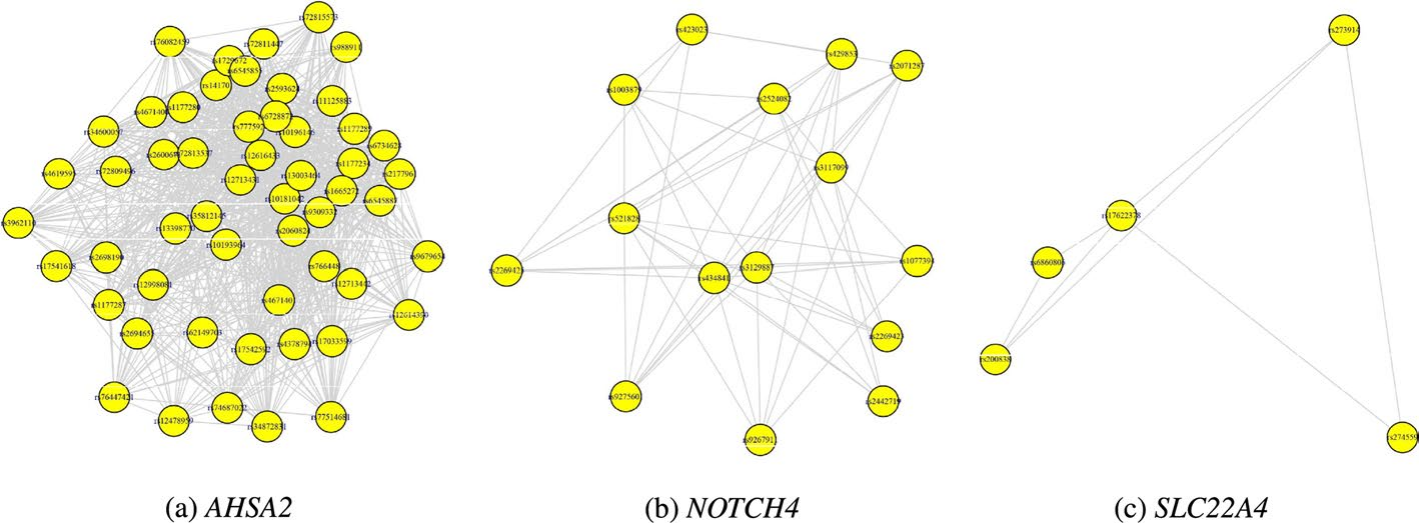
Within-gene bivariate synergy networks (“Within-gene network analysis” section). Number of SNPs varied among genes

**Fig. 3:**
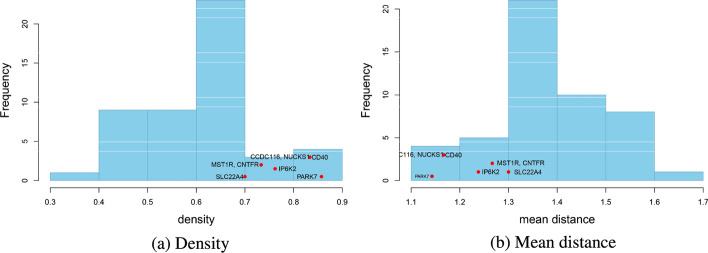
Frequency distributions of within-gene network properties. **a** Density, **b** mean distance, and transitivity (see Additional file 3: Figure S2c). Red dots show top genes(*PARK7, CCDC116, CD40, NUCKS1,IP6K2, CNTFR, MST1R, SLC22A4*) ranked by density. The same genes are highlighted for **b** **(mean distance)**, and additional file 3: Fig S2**c** **(transitivity)**. See Additional file 1: Table S1

**Fig. 4:**
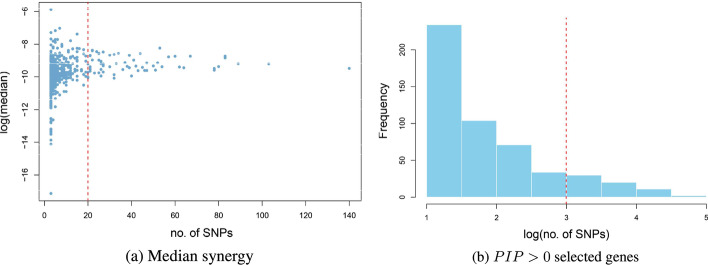
SNP-gene properties. **a** The log median synergy (*Syn*) against the number of SNPs per $PIP>0$ main effect gene (i.e. selected gene); **b** frequency of gene sizes for main effects genes identified via $PIP>0$; The red vertical line indicates gene size of 20. For the histogram in **b**, a log transformation of the number of SNPs was taken for better presentation

Figure 2a–c display the within-gene networks between SNPs that were annotated to *AHSA2, NOTCH4* and *SLC22A4* specifically. These exemplifying genes were picked up by the adopted Bayesian modelling strategy (interaction $PIP>0$) and suggest that gene size (number of SNPs) was not the determining factor for epistasis detection. In general, within gene networks differed by genes (Fig. 3). Genes such as *PARK7, CD40, NUCKS1, MST1R* and *SLC22A4*, known to be associated with IBD (CD or UC) showed higher-end densities (Fig. 3).The number of SNPs mapped to a respective gene varied from 140 SNPs for the HLA-K gene to 2 SNPs for genes such as *ZNF7* and *TRPT1*. The majority of genes had less than 20 SNPs, distance-wise mapped to them (Fig. 4b). There was no obvious relationship between the number of SNPs per main effects gene ($PIP>0$) and the corresponding median synergies (Fig. 4a).

**Fig. 5:**
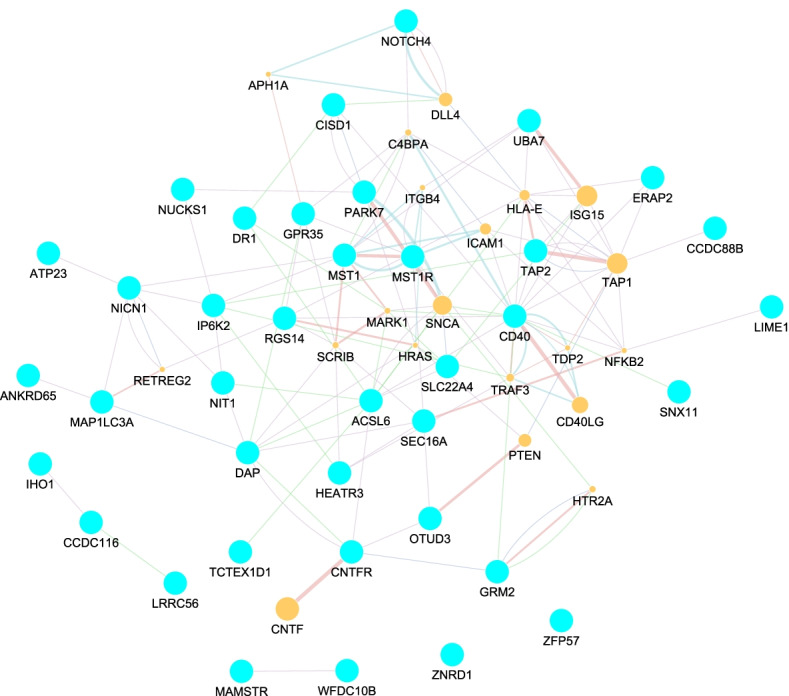
Gene interaction network for main genes effects as in Additional file 2: Table S2. Light-blue dots refer to interrogated genes; orange dots are genes retrieved from the GeneMANIA databases further shaping the interaction network.

**Fig. 6:**
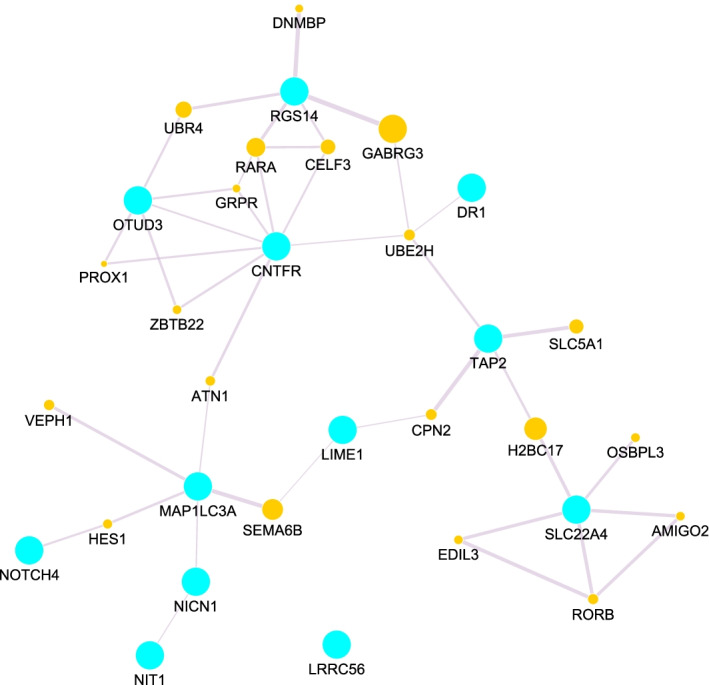
Gene interaction network for genes identified via interaction $PIP>0$ (Table 1). Light-blue dots refer to interrogated genes; orange dots are genes retrieved from the GeneMANIA databases further shaping the interaction network. For PPI network, see Additional file 3: Figure S1

Genes with a marginal or interaction PIP greater than zero were used to construct interaction networks in GeneMANIA, searched on 7th December 2021. For Fig. 5, the search term was the list of all genes with PIP greater than 0 from the main effects model (see Additional file 2: Table S2). For Fig. 6, the search term was the unique set of genes whose interaction had a PIP greater than zero (Table 1). The objective was to retrieve novel connections between our own identified genes and other genes given known interaction information in curated databases.

**Table 1:** Second-order gene–gene interactions and their respective posterior inclusion probabilities (PIP)

Gene 1	Gene 2	PIP
*LINC01475*	*NIT1*	0.0263
*JAZF1.AS1*	*TAP2*	0.0090
*LIME1*	*NICN1*	0.0075
*LRRC56*	*OTUD3*	0.0071
*AHSA2*	*NOTCH4*	0.0025
*CNTFR*	*EIF2S2P3*	0.0025
*MAP1LC3A*	*RGS14*	0.0025
*CDC37P1*	*OIP5.AS1*	0.0015
*CDC37P1*	*SLC22A4*	0.0005
*OIP5.AS1*	*SLC22A4*	0.0005
*DR1*	*MAP1LC3A*	0.0003

Furthermore, we searched for external evidence for association of our selected genes with IBD, Crohn’s Disease (CD) or Ulcerative Colitis(UC) using DAVID and DisGeNET. Genes *SLC22A4, OTUD3, PARK7, NOTCH4, GPR35, DAP, UBA7, MST1, MST1R, CD40, TAP2, NICNI, RGS14, LINCO1475, GBAP1, NUCKS1, HCG23, CCDC88B, HEART3,* and *ERAP2*, have been previously associated with IBD, CD or UC. Of the genes involved in 2-way interactions (Table 1), seven genes namely *LINCO1475, TAP2, RGS14, OTUD3, SLC22A4, NICN1* and *NOTCH4* have already been associated with IBD, CD or UC. From the network (Fig. 6), genes *MAP1LC3A, UBR4* and *CNTFR* are attractive for further investigation. Their degree (number of connected edges) and nature of connections in which they are involved warrant in-depth investigation of their potential roles in IBD pathology. For instance, *MAP1LC3A* has degree 5, while *CNTFR* has a degree of 8, with the both genes being central to two clusters of genes in the network. Also, *MAP1LC3A* has been implicated in related diseases e.g. in cancers of the gastro intestinal system [45, 46]. Gene *CNTFR* has been implicated in rheumatoid arthritis, a chronic inflamatory disorder [47], and *UBR4* in anorectal malformations and stomach cancer [48, 49].

## Discussion

High-throughput technologies have facilitated multi-omics profiling of individuals. Since the first complete sequence of the human genome, several genome-wide association studies comparing cases and controls have emerged. These studies have been successful to obtain a better understanding of biological underpinnings of complex diseases such as Inflammatory Bowel Disease (IBD), a highly prevalent disease that is characterized by chronic inflammation of the gastrointestinal tract [50]. According to DisGeNet and the NHGRI-EBI GWAS Catalog, over 170 genes are reported to be associated with inflammatory bowel diseases. Despite the appreciable number of GWAS findings, only a few of these have shown immediate impact in clinical practice. This is not surprising. GWAS only offer one view of the complexity of an individual’s health. Interactome analyses offer another view. The interactome refers to the entire complement of interactions between DNA, RNA, proteins and metabolites within a cell. In this work, we have focused on one particular type of interactions, namely “DNA–DNA” interactions, using genetic markers from GWAS, in particular SNPs, as vehicles. The context is thus genome-wide association interaction studies (GWAIS), building on GWAS and aiming to identify interacting SNPs in relation to a phenotype of interest.

Several reviews exist on GWAIS. These focus on the definition of epistasis [5, 6], on the vast amount of analytic approaches that exist [3, 6, 51] on the difference between capturing and detecting epistasis [52] or on how to adapt the analysis workflow to increase our belief in statistical interactions with SNPs and to maximize translation opportunities [4], amongst others. The latter reference highlights the many issues that still exist for GWAIS analyses, including replication issues at the SNP level or interference by linkage disequilibrium patterns in the data. Gene-level epistasis analysis may be better suited towards increased interpretability and replication across studies. Also, they may be the road to travel by for epistasis detection with GWAS SNP data when millions of SNPs need to be screened. In practice, such enormous datasets require adopting filtering or dimensionality reduction approaches. These may involve the incorporation of prior biological knowledge (for instance, restricting the search space to SNP-level interactions that involve known gene-level biological interactions) [53], or a transformation of SNP-level to gene-level analysis via tissue-specific SNP induced estimates of gene expression [54]. To our knowledge, one of the first gene-level epistasis analysis methods, starting from GWAS SNPs and creating gene-level summaries, is gene-based MDR [55]. For each gene, the best within-gene SNP interaction model is considered to be a gene-level binary predictor for the trait and serves as input to a second run of MDR to find the best gene-level interaction model.

In this study, we identify epistasis signals via GWAS data by creating SNP sets allocated to the same gene and endowing the SNP sets with a network structure. The network structure allows information to be diffused over the corresponding graph and summarizing the SNP sets with diffusion kernel PCs (for instance, the first such component for each gene). The derived gene summaries serve as input to gene-level interaction models. In what follows, we motivate the components of this workflow and show how elements may be customized to accommodate different application settings.

Several SNP-to-gene mapping strategies exist. To exemplify the analysis workflow, we chose the most commonly used mapping method based on genetic distance with the FUMA software. Adopting such a genomic proximity mapping requires making additional choices of the maximum allowable distance (f.i., taking the closest gene or genes within a number of kbps of the index SNP). Alternatively, SNPs may be mapped to genes in a functional way. One example is eQTL mapping. Depending on the mapping strategy, several useful SNPs may be eliminated from the analysis. On the other hand, different opportunities to define a graphical structure between the SNPs annotated to the same gene may emerge. For instance, with genomic proximity mapping the sequence of SNPs on the genome can be used (LinearNet). Network edges may also be defined on the basis of linkage disequilibrium patterns between SNPs (LDNet). These approaches have been investigated before in the context of Type II diabetes [56]. With eQTL functional mapping, SNPs mapped to the same gene may be connected to each other when they act as each other’s modifier in an eQTL epistasis relationship. The latter would imply the creation of SNP-set modules that are not disease-trait informed, but that can be regarded as “functional modules”.

The use of entropy measures in GWAS and epistasis research is not new. For instance [57] considered Ŕenyi entropy based single locus and two-locus association testing. A few years later, entropy-based test statistics for gene-gene interaction studies were reviewed in [58]. This study highlights the wide diversity of such measures. It should be noted that entropy-related concepts may be used differently by different authors as is the case for “information gain” (for example: [59–61]). Also, entropy-based measures may capture joint multi-locus effects [61] or purely interactive effects (no influence of main effects) as is the case in [62]. Furthermore, most software tools producing entropy based estimators require complete data. For this reason, we included an additional imputation step in the analysis protocol. That is, for available SNPs in the data, missing genotypes were imputed via k-nearest neighbors. More elaborate imputation strategies based on haplotypes or linkage disequilibrium exist and are commonly used in GWAS context but were not considered in this pilot analysis workflow. Alternatively, apart from being useful in testing, entropy-based measures can also be used in screening as was done in earlier work of ours [59]. In our analysis pipeline, we computed bivariate synergies between SNPs, not between all SNPs as in [59] but only between those that were annotated to the same gene. Furthermore, as only a handful of entropy based estimators for association with a quantitative trait are available (see references in [58]), we chose to discretize our non-binary trait that had been adjusted for confounding variables. It led to within-gene networks of SNPs with edge weights induced by the adopted synergy measure.

We preferred to generate weighted within-gene networks rather than binary networks to avoid specifying a synergy threshold that may well need to be gene-dependent, and to have more refined diffusion of information. The beauty of our implemented strategy over LDNet and LinearNet is that network edges contain phenotype informed information. Furthermore, this approach is not a classical screening step that would reduce the number of subsequent SNP-based epistasis tests, in which case additional adjustments would be needed to account for elevated Type I errors caused by dependent testing and screening analysis stages.

In our approach, inspired by [63], we employed kernel PCA on a “sandwich” kernel matrix which contains a diffusion kernel as “filling”. The dimensions of the “sandwich” kernel are determined by the available number of individuals in the study. In GWAIS we wish to have sufficiently large sample sizes in order to boost the power for epistasis detection. The downside of large data sets is that it imposes challenges when computing principal components. For instance, for the IBD consortium data we used in this study, with over 60,000 individuals, special measures had to be taken when computing the kernels. In particular, we adhered to parallel computing at each stage of matrix multiplication, and also worked on partitions with at least 500GB of memory.

The kernel PCs computed per gene reduce to classical PCs when no association between SNPs within a gene is used. Indeed, in that case, $\beta =0$ and the filling $exp^{\beta L}$ in the “sandwich” kernel reduces to the identity matrix (first term in the Taylor expansion). Using the average of the $exp^{\beta L}$ matrices retrieved from several $\beta$ values is better than using a single fixed $\beta$ value for every gene as it enables the structure of different genes to dictate what the final kernel matrix would be. An area for further research is to better tune the $\beta$ values especially for studies with large sample sizes for which eigen decompositions tend to be computationally demanding in the R software. Although the number of kernel PCs can be chosen using cross-validation, we chose the first kernel PC as main representative for each gene, giving rise to a single score for each gene per individual.

Notably, unlike gene-based MDR, we are not limiting gene summaries to a single SNP-level epistasis model. Rather, in our analysis workflow we possibly use an entire network structure between SNPs allocated to the same gene to summarize the gene. As suggested before, our approach is flexible in that the network structure may use trait information or not. When a within-network structure is absent, the corresponding gene summary boils down to classical (first) principal component derived from the gene set. Using principal components to summarize SNP information within a gene has been used before in gene-level interaction testing and genomewide association settings [64]. In the latter reference, as an alternative, trait information is used while summarizing a SNP set via partial least squares. Whereas [64] the first components are taken as gene summaries, in [65] genes are summarized by principal components that explain at least 80% of the variation. In contrast, in [66], SNP sets mapped to a gene pair are summarized by a so-called Eigen-Epistasis component. It stands for the linear combination of all respective SNP-SNP interactions that is the most correlated with the phenotype. We did not compare our implemented workflow with aforementioned existing methods, as only gene-based MDR also uses within-gene interaction information to derive gene summaries and since (SNP-based) MDR, on which it relies, suffers from several drawbacks as outlined in [67].

Bayesian models have several advantages, perhaps one of the most apparent one is that they naturally accommodate the inclusion of prior biological knowledge about associations. Several classes of such models for epistasis detection exist, including the BEAM model as reviewed and extended in [68]. These models were shown to have rather low computational complexities. Here, we used gene representative kernel PCs in conjunction with a novel semi-parametric Bayesian model [34], while making inferences about gene and gene-gene interaction effects via Posterior Inclusion Probabilities (PIP), rather than via *p* values. GWAIS *p* values need to be adjusted for a huge number of multiple tests that may exhibit complex dependency structures between them. This imposes unresolved challenges of potentially high false negative rates and at the same time false positive rates that may not be acceptable [69]. It should be noted though that the adoption of Bayesian methods do not necessarily avoid the need for multiple testing correction, as was pointed out in [70]. In either case, in the current workflow, we took a PIP threshold of zero meaning that any gene or gene-gene interaction with a PIP strictly larger than zero was considered to be of predictive value to the phenotype and was included in downstream analyses. By no means should PIPs be interpreted as measures of association strength. Another motivation to work within a Bayesian paradigm is that in our workflow, only a relatively small number of variables needed to be mined (theoretically, as many variables as there are genes). Often, the advantages of some classes of Bayesian models in epistasis research are downplayed by the necessity to first filter the data and to reduce its dimensionality. Working with gene summaries as we have defined them naturally deals with this issue.

The final step of the analysis workflow involved interpretation of findings. All epistasis results were visualized in a gene interaction network and analyzed using network theory. The network may be the direct result of inferred interactions, but may also be the result of consulting external resources with “interaction” information. One such resource is GeneMANIA. By entering genes that were analytically identified as putative gene-gene interaction and/or as putative main effect (via $PIP>0$), GeneMANIA will build an entire network around them. Alternatively, identified genes can be propagated on a molecular network such as inBio, STRING, as in [71]. This bigger context may highlight interesting genes that were not directly identified via our epistasis detection models. It may highlight novel disease gene clusters and shed additional light on disease-related biological mechanisms. Notably, several molecular interaction databases exist each of them having differential performance, for instance in retrieving relevant disease genes [71]. Hence, experimental validation of promising results remains inevitable but may not always be feasible or accepted without replication evidence. Unfortunately, currently, there is no consensus in what replication means or should mean in the context of GWAIS [72].

Our analysis workflow applied to IBD spotlighted several known IBD associated genes and identified several gene-gene interactions. In practice, 41% of the 49 genes highlighted by our approach (PIP $>0$) could be traced back to previous reports about associations with IBD (Crohn’s, Ulcerative Colitis). Additional work is needed to investigate indirect links with IBD phenotypes. Comparing networks derived from analytic main effects and epistasis modelling complements the picture. For instance, Figs. 5 and 6 underscored three genes, namely *MAP1LC3A, RGS14* and *CNFTR*. No association evidence between these genes and IBD could be found. Regardless, from a network point of view these 3 genes are interesting and worthwhile to follow-up in an attempt to determine their potential role in IBD pathology.

All of the above shows that the trait-informed dimensionality reduction step in our novel epistasis detection analysis workflow enhances the detection of gene-gene interaction effects and can detect genes associated with the phenotype. It fosters novel think tank paths to spur medical innovations. Additional optimizations at multiple layers of the analysis protocol are possible (as discussed) and are believed to further enhance the performance of our approach.

## Supplementary information

**Additional file 1. Table S1:** Within gene network properties.

**Additional file 2. Table S2:** Main effects from model.

**Additional file 3.** supplementary figures.

## Data Availability

Data on IBD can be accessed via the International IBD Genetics Consortium (www.ibdgenetics.org). For this project, data were obtained through a Memorandum of Understanding with a representative of the consortium from the University of Liège and KU Leuven. Any interested party can access the data through a similar procedure.

## Code availability

The code necessary to reproduce this article’s results and analyses is available on GitHub at: https://github.com/awalakira/kPCAepistasis.
