# Supplementary material for: Detecting gene–gene interactions from GWAS using diffusion kernel principal components
Source: BMC Bioinformatics. 2022 Feb 1;23:57. doi: 10.1186/s12859-022-04580-7 (PMC8805268; doi:10.1186/s12859-022-04580-7)
Supplement: Supplementary file 3 — Additional file 3. supplementary figures. [file 12859_2022_4580_MOESM3_ESM.pdf]

Appendix figures for:

Detecting gene-gene interactions from GWAS using diffusion kernel principal components

Andrew Walakira[2*], Junior Ocira[1], Diane Duroux[1], Ramouna Fouladi[1], Miha Moškon[3], Damjana Rozman[2], and Kristel Van Steen[1,4]

[1]BIO3 - Laboratory for Systems Genetics, GIGA-R Medical Genomics, University of Liège, Liège, Belgium [2]Centre for Functional Genomics and Bio-Chips, Institute for Biochemistry and Molecular Genetics, Faculty of Medicine, University of Ljubljana, Ljubljana, Slovenia [3]Faculty of Computer and Information Science, University of Ljubljana, Slovenia [4]BIO3 - Laboratory for Systems Medicine, Department of Human Genetics, KU Leuven, Leuven, Belgium

[*] corresponding author
E-mail: andrew.walakira@mf.uni-lj.si

1 Supplementary Figures

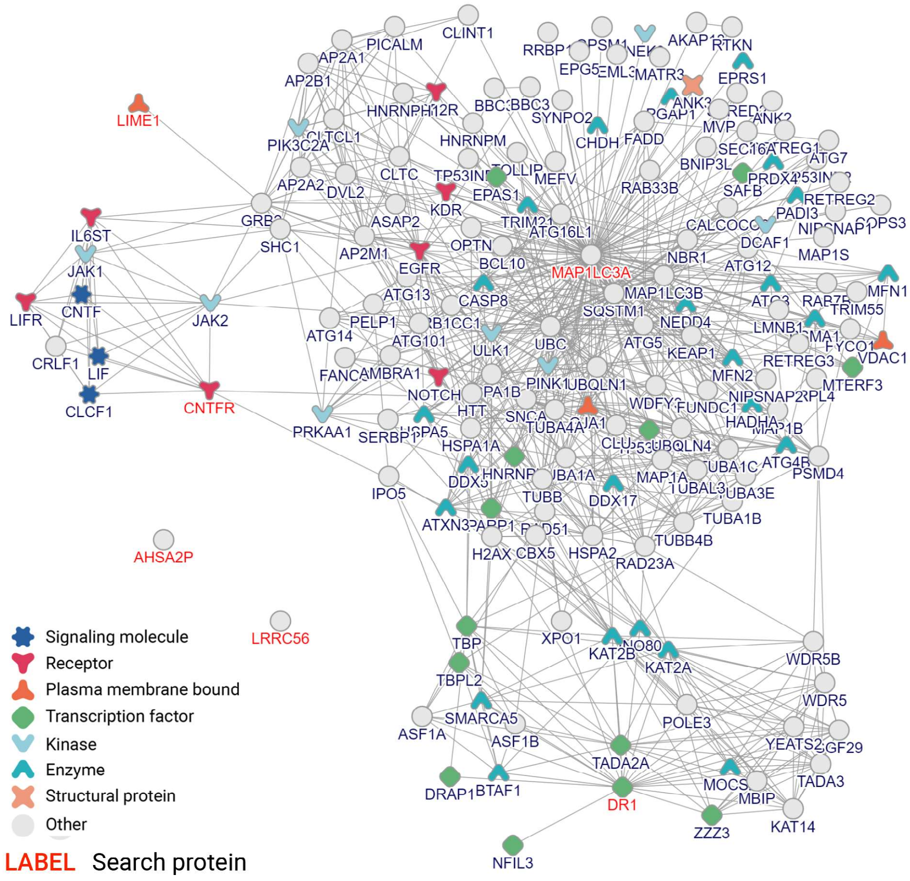

Supplementary Figure 1: Protein-protein interaction network for genes identified via interaction $PIP > 0$ (Table 1). *MAP1LC3A* and *CNTFR* are key hubs

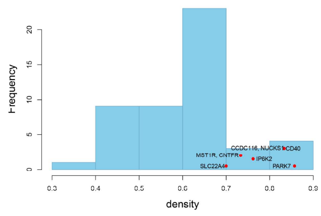

(a) Density

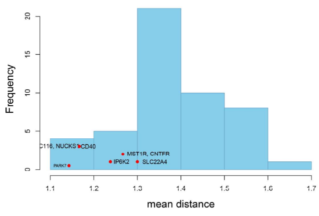

(b) Mean distance

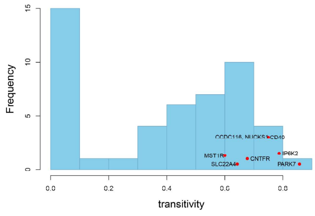

(c) Transitivity

Supplementary Figure 2: Frequency distributions of within-gene network properties. (a) density, (b) mean distance and (c) transitivity. Red dots show top genes(*PARK7, CCDC116, CD40, NUCKS1,IP6K2, CNTFR, MST1R, SLC22A4*) ranked by density. The same genes are highlighted for (b) and (c). See Supplementary Table 1

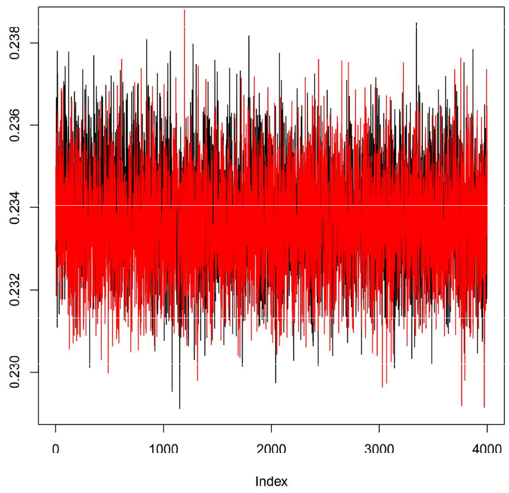

(a) Trace plot

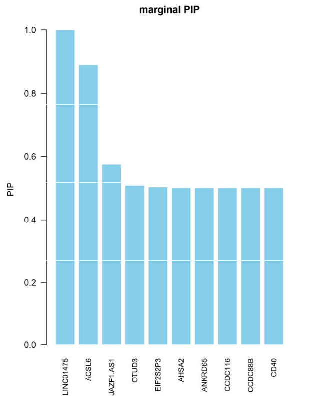

(b) marginal PIP for top genes

Supplementary Figure 3: The trace plot (on the left) shows that our model converges. On the right, the bar graph shows the top marginal PIP. Forty nine genes were selected to test for interaction
